# A Two-Stage Transferred Cold Atmospheric Plasma as a Unique Therapeutic Strategy for Targeting Colon Cancer Stem Cells

**DOI:** 10.34172/apb.2024.041

**Published:** 2024-03-20

**Authors:** Abolfazl Soulat, Taghi Mohsenpour, Leila Roshangar, Hamid Naghshara

**Affiliations:** ^1^Department of Atomic and Molecular Physics, Faculty of Sciences, University of Mazandaran, 47416-13534, Babolsar, Iran.; ^2^Stem Cell Research Center, Tabriz University of Medical Sciences, 5166614766, Tabriz, Iran.; ^3^Faculty of Physics, University of Tabriz, 5166616471, Tabriz, Iran.

**Keywords:** Two-stage transferred cold atmospheric plasma, Cancer cells, Colon cancer stem cells, Apoptosis

## Abstract

The study examines the induction of apoptosis in colon cancer stem cells (CCSCs) within a 3D culture setting, employing an innovative cold atmospheric plasma (CAP) transmission method known as two-stage transferred cold atmospheric plasma (TS-TCAP). TS-TCAP is a partially or fully ionized non-thermal gaseous mixture that comprises photons, charged and neutral particles, and free radicals, which has gained traction in biomedical applications such as cancer therapy. TS-TCAP impacts CCSCs via a continuous, two-step transport process, facilitating the efficient delivery of reactive oxygen and nitrogen species (RONS). The key cellular factors of CCSCs impacted by TS-TCAP treatment, encompassing the secretion and expression levels of IL-6 and IL-8, apoptotic cell count, and expression of BAX, BCL-2, and KI-67 proteins, were evaluated using qrt-ELISA, Annexin V, and qrt-PCR procedures, respectively. The outcomes of CCSCs treatment with TS-TCAP reveal a notable rise in the number of apoptotic cells (*P*<0.0001), diminished secretion, and gene expression of IL-6 and IL-8 (*P*<0.0001), accompanied by favorable alterations in BCL-2 and BAX gene expression (*P*<0.0001). Additionally, a notable decrease in KI-67 expression was observed, correlating with a reduction in CCSCs proliferation (*P*<0.0001). As well, this study underscores the anti-cancer potential of TS-TCAP, showcasing its efficacy in reducing CCSCs survival rates. However, further pre-clinical and clinical trials are necessary to evaluate CAP’s efficacy, safety, and potential synergistic effects with other therapies thoroughly. Overall, TS-TCAP presents a promising alternative for CCSCs treatment, pending further investigation and refinement.

## Introduction

 Colorectal cancer (CRC), recognized as a substantial and serious malignancy, holds a prominent position among the top three cancers, both in terms of prevalence and mortality rates.^1^ The metastasis of this type of cancer to distant organs, particularly the liver, stands out as a primary contributor to the mortality associated with this condition.^2,3^ Commonly applied cancer therapies struggle with the difficulty of drug resistance incidence, resulting in the accumulation of cancer stem cells (CSCs) and the reoccurrence of treated tumors.^4^ CSCs, as the underlying factor of phenotypic heterogeneity in diverse cancers, are minor subpopulations endowed with attributes like self-renewal, differentiation, and tumorigenesis.^5^ Moreover, CSCs are acknowledged as the primary contributors to treatment resistance, metastasis, and the recurrence of tumors.^6^ While conventional treatments such as surgery, radiotherapy, and chemotherapy are effective in targeting a substantial portion of the tumor associated with CSCs, they lack the capability to eradicate the specific CSCs responsible for metastasis and tumor recurrence.^7^ Emerging therapeutic methods aim to target various aspects of the tumor microenvironment and destroy drug-resistant tumor cells, especially CSCs.^8,9^ One of the most innovative and crucial technologies in cancer therapy is plasma-physical systems, specifically cold atmospheric plasma (CAP).^10^

 CAP is characterized as a non-thermal gas mixture, either partially or fully ionized, comprising a combination of charged and neutral particles, including electrons, ions, radicals, and high-energy photons.^11^ Operating at atmospheric pressure and ambient temperature, CAP generates reactive oxygen and nitrogen species (RONS) through interactions with ambient air or specific soluble substances.^12^ Various research results indicate that CAP’s biological impacts are mainly linked to the generation of RONS within the plasma.^13^ Dielectric barrier discharge (DBD) and atmospheric pressure plasma jet (APPJ)^14^ are recognized as the most common and important sources of CAP in medical applications such as sterilization,^15^ wound healing,^16,17^ dentistry,^18^ destroying microorganisms,^19^ and cancer therapy.^20^ Lately, transferred cold atmospheric plasma (TCAP) has become a prominent technique for producing CAP.^21^ In this method, an APPJ is ignited as transferred plasma from an ignited primary CAP source, such as a DBD at the downstream terminal of a thin, lengthy plastic pipe.^22^

 Biologically, two perspectives have been identified on using the CAP in medical applications.^23,24^ The first is direct exposure to plasma, in which plasma directly contacts the biological target, and thus all the plasma-generated agents activate on the cell or tissue.^25^ Conversely, when a medium or solution is affected by the plasma, it is considered an indirect application.^26^ The first pioneering study assessing CAP potency for biomedical applications was conducted for more than a decade (1995–2004).^11,27^ Nevertheless, the most thrilling application of CAP in the fields of medicine and biology is its potential to kill and treat cancer cells and stem cells.^28,29^ Several studies have verified the antitumor features of CAPs on various tumor or solid malignant cells.^30-35^ Also, CAP has the remarkable capability of suppressing tumor growth and inducing destruction or apoptosis in a range of melanoma cells, but it could also hinder the migration and invasion of these cells.^36-39^ Furthermore, CAP enhances the sensitivity of tumor cells to chemotherapy drugs, leading to highly effective antitumor effects with minimal toxicity.^40^ Additionally, numerous studies have verified that the synergy between CAPs and nanoparticles has substantial anti-tumor impacts.^41^

 The objective of this experimental investigation is to explore the effects of two-stage transferred cold atmospheric plasma (TS-TCAP) on the survival and apoptosis of cultured colon cancer stem cells (CCSCs) in a three-dimensional culture environment. The research specifically emphasizes diverse parameters related to apoptosis, cell survival, inflammatory factors, and the biocompatibility of CCSCs. This encompasses the analysis of gene expressions, including BAX, BCL-2, IL-6, IL-8, and KI-67.

## Methods and Materials

 This study aimed to explore the effects of TS-TCAP on the survival and apoptosis of CCSCs in a 3D culture setting. To accomplish this objective, the research was divided into four distinct phases. In the initial phase, a kilohertz variable-frequency AC power supply was designed and constructed to generate and optimize a TS-TCAP infused with argon and helium gas. The characteristics and properties of the transferred TS-TCAP, such as voltage, current, frequency, gas flow rate, and the type of generated reaction, were documented and recorded. In the second phase, CCSCs were cultured in a 3D culture medium. Thirdly, CCSCs cultured in three-dimensional environments were subjected to direct exposure to the TS-TCAP. In the final phase, plasma-treated cells were subjected to biological, and histological analyses, including RT-PCR for gene expression analysis. Furthermore, ELISA methods were employed to assess oxidative pathways. Biochemical and flow cytometric analyses were conducted, and the expression of proteins and enzymes associated with apoptosis, such as BAX and BCL-2, was verified using apoptosis annexin V evaluation kits. In this research, an MTT assay was conducted to assess the viability of CCSCs after direct exposure to TS-TCAP for 3 and 5 minutes.

###  TS-TCAP

**Figure 1:**
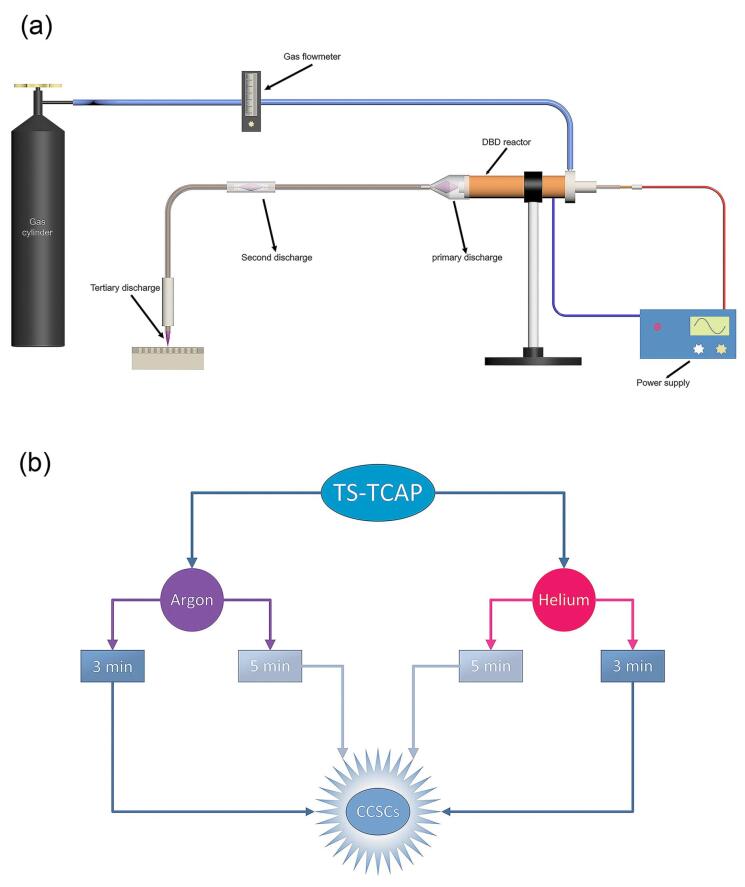
(a) Schematic diagram of the structure of TS-TCAP, (b) Schematic diagram of the experimental group design

 This study introduces an innovative approach for the secure transfer of CAP. The experimental set-up, illustrated in Figure 1a, involves an APPJ generated at both ends of flexible plastic tubes. The key feature is a significant reduction in initial voltage at the tube extremities. The configuration includes three main parts: plasma zone, power supply, and diagnostic/control system.

 The plasma zone comprises a primary discharge chamber, a first transmission tube with a copper core, a second discharge chamber, and a second transmission tube with a copper core, from which the APPJ emerges. The primary discharge occurs in a coaxial double-barrier dielectric barrier discharge (DB-DBD) reactor with outer (copper tube, 24 mm in diameter and 2 mm in thickness) and inner (copper wire, 2.5 mm in diameter) copper electrodes separated by a closed-end quartz tube (4 mm in diameter and 1 mm in thickness) with a polytetrafluoroethylene (PTFE) layer (15 mm in diameter). The inner electrode is connected to a high-voltage power supply, and the outer electrode is grounded. A funnel-shaped Pyrex tube and a 5-meter transmission tube with a copper core (0.2 mm in diameter) facilitate plasma transfer. Between the two transmission tubes, a quartz tube (5 mm in diameter, 1 mm in thickness, and 5 cm in length) separates two copper wires. When a high voltage is applied to the internal electrode of the DBD reactor, it generates a potent electric field between the internal and external electrodes. Since the plasma’s performance is significantly influenced by nearby conducting bodies,^42^ this electric field induces the creation of plasma filaments between the end of the internal electrode and the beginning of the copper wire of the initial transfer tube. After the potential is applied to the DBD reactor, the first copper wire generates a potent electric field at its downstream end. This field leads to the ionization of gas particles and facilitates the primary discharge initiated within the DBD reactor, occurring between the two copper wires of the first and second transfer tubes and within the quartz connector as a secondary discharge. Similarly, the copper wire of the second transmission tube, influenced by the potential of the copper wire of the first transmission tube, generates an electric field at its downstream end. This leads to the formation of an APPJ originating from the end of the second transmission tube, serving as the third and final discharge.

 The power supply is an 800 W half-bridge followed by a 1:60 ferrite transformer, producing an 18 kVp-p output voltage limited to 500 mA. Optical emissions diagnostics use an Ocean Optics USB4000 spectrometer, while electrical data collection involves a Tektronix TDS2024D oscilloscope with an AOP-10 HV probe and a conventional 10X probe. Charge measurement employs a 2.7 nF (nano-farad) capacitor with a 1000 V rating, and the voltage at the second copper wire end is measured using a 1:1000X probe. Charge value is calculated by multiplying capacitor capacitance by voltage drop, recorded with a 1:10X probe.

###  Cell culture in matrigel

 CCSCs, isolated from the HT29 cell line, were acquired from the NCBI of Iran Pasteur Institute cell bank. These cells were cultured in RPMI-1640 media supplemented with 10% heat-inactivated fetal bovine serum (FBS), penicillin (100 U/mL), and streptomycin (100 µg/mL). The cells were incubated in a humidified 37 °C incubator with 95% air and 5% CO2. The study focused on investigating the direct impact of TS-TCAP on the survival and apoptosis of CCSCs in a 3D culture.

 For plate preparation, 200 μL of Matrigel matrix (10 mg/mL) was added to each well of a pre-chilled 48-well plate. After spreading the Matrigel matrix with a pipet tip, the plate was incubated at 37 °C for 30 minutes to allow gel formation. Subsequently, CCSCs were seeded in 48-well plates at a density of 2 × 10^4^ cells/well (Bio-tech, Shanghai, China) using a culture medium containing 500 µ Dulbecco’s modified eagle medium (DMEM), 10% FBS, 100 U/mL penicillin G, and 100 mg/mL streptomycin (Gibco, USA) for 14–18 hours. Following this, 250 µL of the culture medium was replaced with an equivalent volume of serum-free CSC medium. This medium comprised DMEM/F12 (Gibco), recombinant human epidermal growth factor (rhEGF, 20 ng/mL; Sigma, USA), basic fibroblast growth factor (bFGF, 20 ng/mL; Upstate, USA), B27 (1, Gibco), leukemia inhibitory factor (LIF, 10 ng/mL; Sigma, USA), and insulin (4 U/L; Sigma), with or without vincristine (5 ng/mL; Hualian Pharmaceutical Co., Ltd., Shanghai, China). The same procedure was repeated for subsequent experiments.

###  Study design

 The methods, procedures, and experiments for cell handling received approval from the Ethics Committee of Tabriz University of Medical Sciences. The cultured cells were randomly divided into two primary groups, as illustrated in Figure 1b: Group A underwent direct exposure to argon TS-TCAP for durations of 3 and 5 minutes; Group B was directly exposed to helium TS-TCAP for 3 and 5 minutes.

###  In-vitro assay of cell viability

 The MTT technique was employed to assess both the direct impacts of TS-TCAP and the effects of argon and helium gases on the viability of CCSCs. The MTT test for each sample was analyzed in triplicate. In each well of 96-well microplates (Nunc, Roskilde, Denmark), 5 × 10^3^ cells were seeded onto a sterilized scaffold. Following 24, 48, and 72 hours of cultivation, 50 µL of MTT solution was added to each well, and incubation took place in a 5% CO2 incubator at 37 °C in a humid environment for 24 hours to allow bone marrow mesenchymal stromal cells (BMMSCs) to adhere to the well surfaces. Wells containing CCSCs and the standard culture medium served as controls. Subsequently, the culture medium and any remaining MTT solution were removed from the wells, and 100 µL of DMSO was added to dissolve the formed formazan crystals. Absorbance was then measured at a wavelength of 570 nm using an ELISA plate reader (Model XYZ, Roche Applied Science, Indianapolis, USA). The survival percentage of the different groups was compared to that of the control group.

###  RT-PCR

 The gene expression levels of ATP-binding cassette (ABC) transporters responsible for vincristine efflux, specifically multidrug resistance protein 1 (MDR1) and multidrug resistance-associated protein 1 (MRP1), were determined through RT-PCR analysis. Total RNA was extracted from both adherent nonsphere-forming cells and tumor spheres of U87 cells using Tripure reagent (Roche Co., Switzerland), following the manufacturer’s protocol. RT-PCR was conducted using the TaKaRa RNA PCR kit 3.0 (TaKaRa, Japan). The glyceraldehyde-3-phosphate dehydrogenase (GAPDH) gene was amplified as an internal control, with each gene undergoing 29 cycles of amplification. Subsequently, each RT-PCR aliquot was electrophoresed in a 1.8% agarose gel containing 0.5 mg/mL ethidium bromide.

###  Flow cytometry

 The conditions conducive to inducing apoptosis were assessed through dual staining with Annexin V-FITC and Propidium Iodide (PI), followed by flow cytometry analysis. Initially, cells were seeded into 6-well plates at a density of 2.5 × 10^5^ cells per well. Subsequently, following trypsinization, cells from all experimental groups were stained with Annexin V-FITC and PI for 15 minutes at 4 °C in the dark. Finally, the stained cells were examined for the percentage of apoptotic cells utilizing a Partec flow cytometer and FloMax software.

###  Measurement of IL-6 and IL-8

 IL-6 and IL-8 levels were quantified using the Quantikine ELISA kit (Human CXCL8/IL-8, cat. no. D8000C; human IL-6, cat. no. D6050; R&D Systems, Inc.). The cell-free culture supernatant from each well in all experimental groups was collected, incubated, and its optical density (OD) was measured at 450 nm. The assay is capable of detecting cytokines at concentrations as low as 5 ± 7 pg/mL.

###  Transmission electron microscopy (TEM)

 Secondary spheres, originating from single CSCs derived from primary spheres, were cultured in DMEM supplemented with 10% FBS. These spheres were first fixed in 2.5% glutaraldehyde and subsequently in 1% osmium tetroxide. Following a dehydration process using ethanol and acetone gradients, the samples were embedded in Epon812 resin.

###  Statistical analysis

 Diagrams related to the plasma data were drawn using Origin 2022 (Origin Pro software, Origin Lab Corporation, Northampton, Massachusetts, USA). Furthermore, biological data from all experiments were presented as the mean ± standard deviation (SD). The choice of statistical tests depended on the type of experiment and may have included one-way ANOVA with multiple comparisons, conducted using GraphPad Prism 9.0 (GraphPad Software, San Diego, CA, USA). A significance threshold of *P* < 0.01 was used when comparing treatments to the control.

## Materials

 The specifications of the materials utilized in this study are outlined in Table 1.

**Table 1:** Material specifications utilized in this study

**Materials**	**Specifications**
Argon gas	Sepehr Gaz Kavian, Tehran, Iran
Helium gas	Sepehr Gaz Kavian, Tehran, Iran
Polytetrafluoroethylene polymer (PTFE)	Pars Polymer, Tehran, Iran
Quartz tube	Lianyungang Liaison Quartz, LianYungang, China
Copper electrodes	Mehr Asl, Tabriz, Iran
Plastic tube	Golden Pipe, Isfahan, Iran
Matrigel matrix	Corning, Arizona, USA
48-well plates	Bio-tech, Shanghai, China
96-well microplates	Nunc, Roskilde, Denmark
Streptomycin	Gibco, USA
DMEM/F12	Gibco, USA
Recombinant human epidermal growth factor	Sigma, USA
Basic fibroblast growth factor	Upstate, USA
B27	Gibco, USA
Leukemia inhibitory factor	Sigma, USA
Insulin	Sigma, USA
Vincristine	Hualian Pharmaceutical Co., Ltd., Shanghai, China
ELISA plate reader	Model XYZ, Roche Applied Science, Indianapolis, USA
RNA PCR kit 3.0	TaKaRa, Japan

## Results

###  TS-TCAP

**Figure 2:**
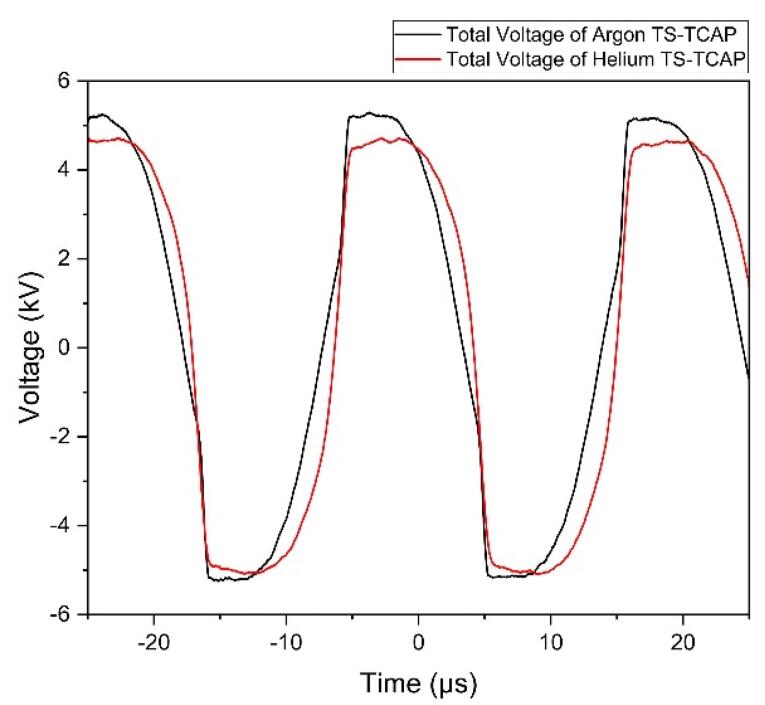
Voltage waveform of the whole plasma system, the end of the second copper wire, and the across the capacitor for: (Black) argon TS-TCAP, and (Red) helium TS-TCAP

 In this study, we employed a novel and unique method to deliver plasma-effective elements (RONSs) to expose CCSCs and investigate their effects on the survival and apoptosis of CCSCs. This new method, used for the first time, is known as plasma two-stage transfer, leading to the induction of an APPJ at the end of the first and second transfer tubes, which we refer to as TS-TCAP. The TS-TCAP described here consisted of three plasma discharges (primary, secondary, and tertiary) using injected argon and helium gases. These discharges were connected by two thin copper wires placed inside thin plastic tubes. The primary discharge was created as the main source of plasma induction and transfer in the DBD reactor. This discharge between the end of the central PTFE and the beginning of the prime copper wire appeared as plasma filaments. The secondary discharge was formed inside a small glass connector that was responsible for connecting the two transfer tubes. In this stage of plasma transfer, similar to primary discharge, plasma filaments were formed between the ends and the beginning of the first and second copper wires. Finally, the tertiary discharge was launched in the form of APPJ from the downstream end of the second transfer tube towards the cultured cells as the final TCAP. To achieve the TS-TCAP composed of argon and helium through these three stages, we applied peak-to-peak voltages of 10.89 kV and 9.78 kV, respectively, to the DBD reactor with a flow rate of 5 L/min. Table 2 shows the electrical characteristics of TS-TCAP. The corresponding frequencies for these voltages were 39.6 kHz and 36.3 kHz, respectively. Figure 2 illustrates the voltage waveform applied to the plasma system for argon and helium TS-TCAP, which was measured concurrently using HV probes.

**Table 2:** Electrical characteristics of argon and helium TS-TCAP

**The parameters**	**argon TS-TCAP**	**helium TS-TCAP**
Total applied voltage (kV)	10.89	9.87
Secondary discharge voltage (kV)	2.334	1.907
Tertiary discharge voltage (kV)	0.367	0.304
Frequency (kHz)	39.6	35.3
Output plasma jet power (W)	0.766	0.819

 The graphs in Figure 2 depict the voltage levels at which all three initial, secondary, and tertiary discharges ignite consistently, optimally, and efficiently. This voltage range was determined based on factors such as the type of gases, the dimensions and distance of the electrodes, the type and thickness of the insulating materials employed (quartz glass and PTFE polymer), and ensuring that the power of the output plasma jet remains below 1 watt to mitigate the potential hazard of electric shock.

 It is worth noting that connecting the HV probe did not affect the ignition of the primary discharge, but the presence of the probe prevented the ignition of the output plasma jet. According to Table 2, a high voltage drop is observed at the end of the second copper wire in comparison to the voltage applied to the DBD. This voltage drop for TS-TCAP of argon and helium is approximately 8.5 kV and 8 kV, respectively, probably due to losses such as ionization losses. This characteristic highlights the safety of TS-TCAP, making it suitable for worry-free use in various medical applications.

 Another reason for the safety of the TS-TCAP used in this research relates to the calculation of the output plasma jet power, which was determined using the following equation:


(1) $$p=f.\;\phi \;V\left(q\right)dq=f.\left[Lissajous\;shape\;area\right]$$

Where V, q, and f represent the voltage of the output plasma jet, the external capacitor charge, and the signal frequency, respectively. According to equation 1, the output plasma jet power is obtained to be 0.766 W and 0.819 W for TS-TCAP of argon and helium, respectively (see Table 2). These power values, which are both below 1W, further support the second reason for the safe use of TS-TCAP in this research.

 Ultimately, reactive species resulting from argon and helium plasma jets were discerned through the optical emission spectrum. Diverse excited species generated by TCAP argon at specific wavelengths encompass Ar I, Ar II, NO, Cu II, O I, O III, N I, N II, N III, N + , N2 + , and O2 + . Additionally, active species identified in TCAP helium at specific wavelengths include He I, Cu I, Cu II, Cu III, O I, O II, N I, N II, N IV, and N2 + .

###  Inflammatory factors

**Figure 3:**
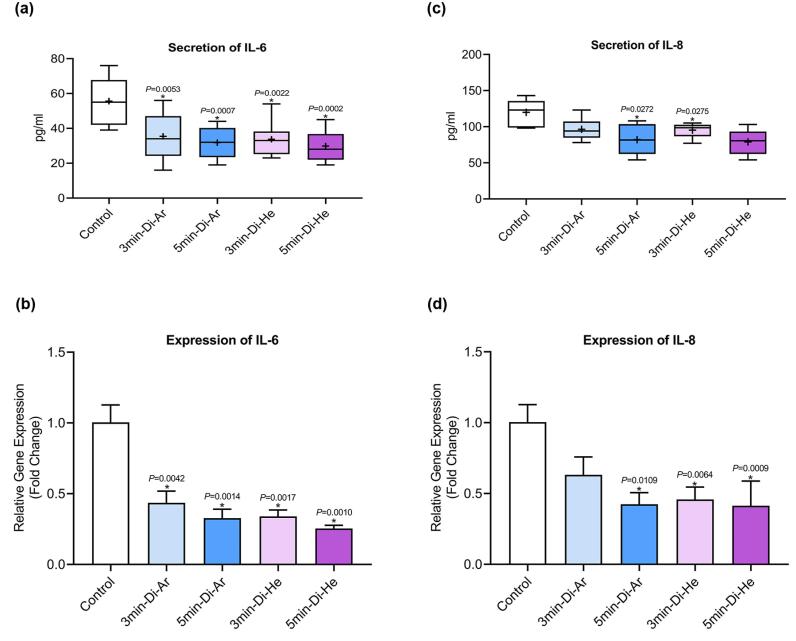
Secretion of (a) IL-6, and (c) IL-8; Expression of (b) IL-6, and (d) IL-8 in experimental and control

 In this study, we investigated the secretion of IL-6 and IL-8 from all experimental groups of CCSCs using ELISA and real-time PCR. The findings indicated that CCSCs released both IL-6 and IL-8 at levels ranging from low to moderate, as shown in Figure 3. Figure 3 depicts a noticeable declining trend in the expression of the studied genes after treatment with both argon and helium gases, and this trend is statistically significant for all the examined genes (*P*< 0.0001). A more detailed analysis of the results reveals that helium gas leads to a more substantial reduction in the expression of the associated genes compared to Argon gas and the overall impact of helium gas treatment is greater than that of argon gas. Furthermore, the duration of irradiation appears to be a significant factor. Increasing the irradiation time from 3 to 5 minutes exerts a more pronounced inhibitory effect on gene expression when helium gas is used for 5 minutes compared to helium gas irradiation for 3 minutes.

###  Apoptosis

**Figure 4:**
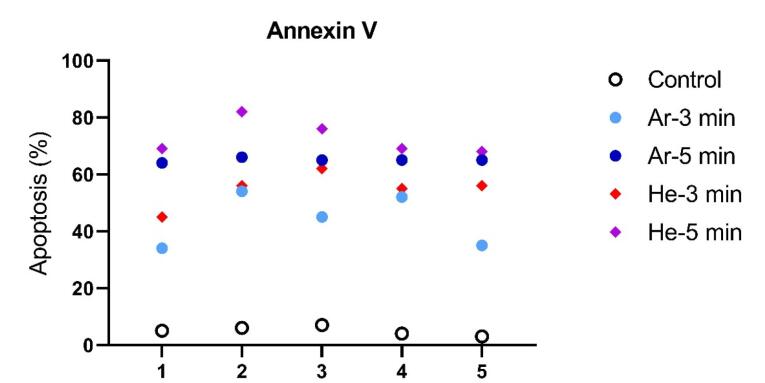
Apoptosis induction in CCSCs: the CCSCs and their exposure to directly TS-TCAP for 3 and 5 min were evaluated for induction of apoptosis using Annexin V by flow cytometry analysis. The percentage of apoptotic cells were presented for each treatment group

 To assess the induction of apoptosis in the experimental groups of CCSCs, Annexin V-FITC was used. All cells were double-stained with Annexin V-FITC and PI, and subsequent analysis was conducted using flow cytometry. The data obtained from flow cytometry analysis were processed with FloMax software to determine the percentages of viable cells (Q3: Annexin V-FITC-/PI-), early apoptotic cells (Q4: Annexin V-FITC + /PI-), late apoptotic cells (Q2: Annexin V-FITC + /PI + ), and necrotic cells (Q1: Annexin V-FITC-/PI + ). The percentage of early (Q4) and late (Q2) apoptotic cells in CCSCs treated with IC50s of Dox (44.98%) and Quer (49.7%) alone and in combination (56.77%), were significantly higher compared to control cells (6.7%) (*P*< 0.0001), as shown in Figure 4.

**Figure 5:**
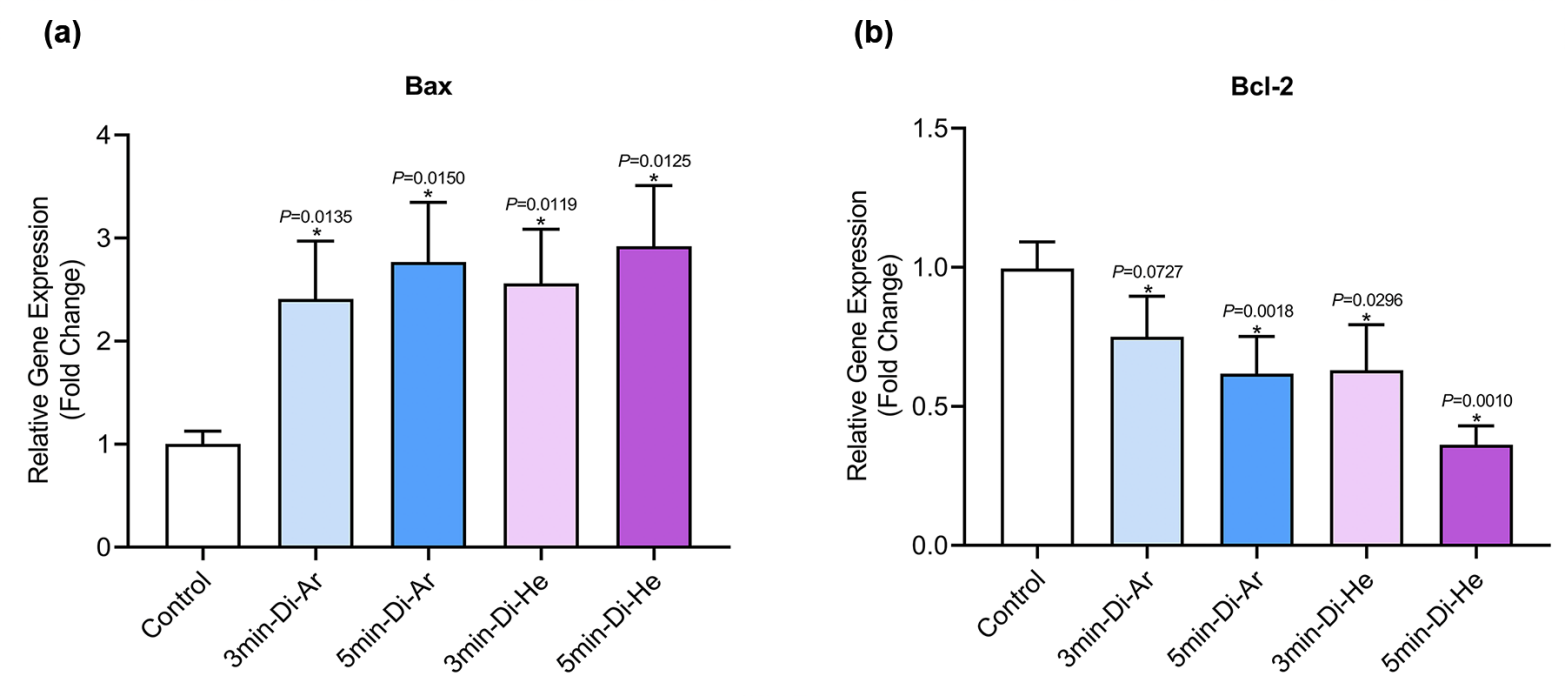
The gene expression results of (a) BAX, and (b) BCL-2 related to CCSCs

 The expression of the BCL-2 gene has shown a significant decrease in all groups when compared to the control group (*P*< 0.0001). This decrease is more pronounced in the groups treated with argon and helium TS-TCAP for 5 minutes. On the other hand, the expression of the BAX gene has significantly increased in all groups compared to the control group (*P*< 0.0001). The ratio between BAX and BCL-2 expression plays a crucial role in determining the fate of cells in response to apoptotic stimulation. An increase in the BAX/BCL-2 ratio decreases cellular resistance to apoptotic stimuli, leading to increased cell death and a reduced incidence of tumors. For a visual representation, please refer to Figure 5.

**Figure 6:**
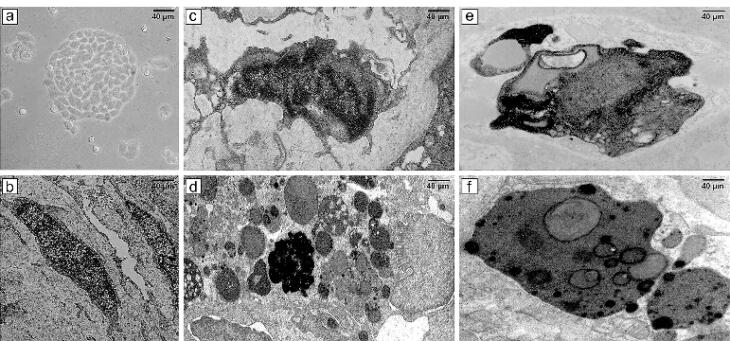
(a) 3D image of CCSCs culture with an inverted microscope; (b) TEM micrograph of a typical cancer stem cell with normal size, nucleus, and organelles; (c) TEM micrograph of morphological alterations of CCSCs after exposure to argon TS-TCAP for 3 minutes; (d) TEM micrograph of morphological changes of CCSCs following exposure to helium TS-TCAP for 3 minutes; (e) TEM micrograph of morphological changes of CCSCs after exposure to argon TS-TCAP for 5 minutes; (f) TEM micrograph of morphological changes of CCSCs following exposure to helium TS-TCAP for 5 minutes

Figure 6 showcases a series of transmission electron micrographs depicting cells under various treatments. In Part (a), a 3D photomicrograph captures the spheroid form of CCSCs aggravations at high magnification using an inverted microscope. Part (b), a normal CSC with typical size, nucleus, and organelles, is observed. This image illustrates a CCSC with typical size and organelles. Part (c) illustrates the effects of argon TS-TCAP for 3 minutes on CCSCs with high chromatin density and chromatin margination characterizing the early apoptotic phase. Part (d) showcases the effects of helium TS-TCAP on CCSCs for 3 minutes. The image reveals high chromatin density and chromatin margination, along with an abundance of apoptotic bodies, plasma membrane blebbing, and secondary lysosomes. Part (e) demonstrated the effects of argon TS-TCAP exposure for 5 minutes. The image highlights high chromatin density, chromatin margination, and numerous apoptotic bodies with secondary lysosomes. Part (f) shows the effects of helium TS-TCAP on the CCSCs for 5 minutes. The nucleus exhibits high chromatin density, chromatin margination, and a significant presence of apoptotic bodies, cell blebbing, as well as numerous secondary lysosomes. Notably, in CCSCs, euchromatin nuclei and extensive organelles, such as the endoplasmic reticulum and clear mitochondria, are clearly observable.

###  Proliferation

**Figure 7:**
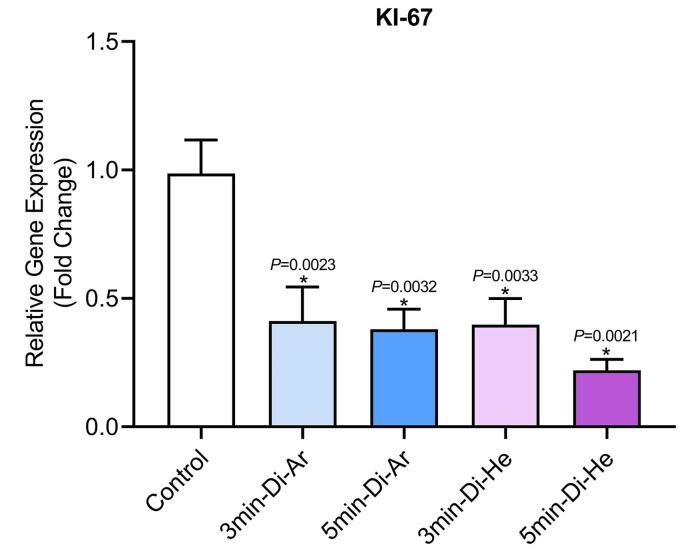
The expression of proliferation-related factor KI-67 in relation to colon cancer

 The results of evaluating the expression of proliferation-related factor KI-67 in CCSCs showed a decrease in all groups (*P*< 0.0001) in KI-67 gene expression when using argon and helium TS-TCAP, as shown in Figure 7. The decrease in gene expression was significant compared to the control group. Also, the duration of radiation was found to be a more important factor than the type of gas used. Additionally, the developed method increased the expression of CCSCs, which increased the expression rate by increasing the irradiation time. The duration of irradiation was also found to be effective. Another important finding was the increased apoptosis of cancer cells after treatment with argon and helium TS-TCAP.

###  In-vitro assay of cell viability

**Figure 8:**
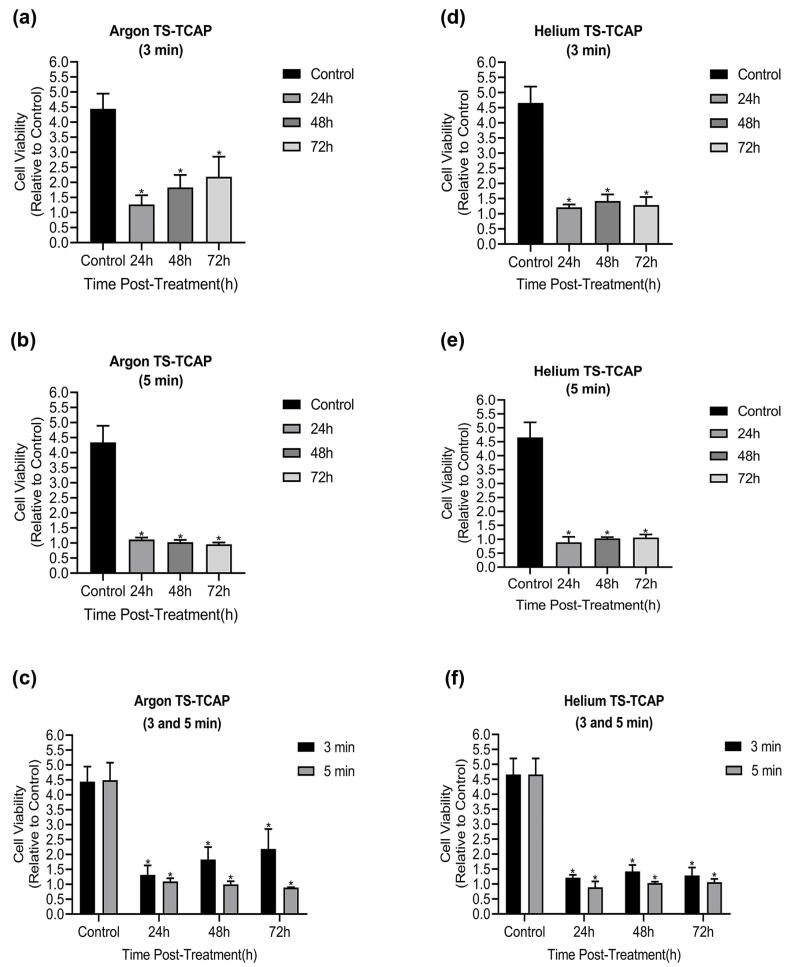
MTT test results: (a) Cell viability of CCSCs after 3 min of direct argon TS-TCAP exposure; (b) Cell viability of CCSC after 5 minutes of direct argon TS-TCAP exposure; (c) CCSC cell viability after 3 and 5 min of direct argon TS-TCAP exposure; (d) CCSC cell viability after 3 min of direct helium TS-TCAP exposure; (e) CCSC cell viability after 5 minutes of direct helium TS-TCAP exposure; (f) Cell viability of CCSCs after 3 and 5 min of helium TS-TCAP exposure

 In this study, the viability of CCSCs was assessed at different time points using the MTT assay when subjected to argon and helium TS-TCAP, as illustrated in Figure 8. Sections (a), (b), and (c) in Figure 8 correspond to the impact of argon TS-TCAP on CCSCs for 3 and 5 minutes. According to the findings, the survival rate of CCSCs significantly decreased when subjected to argon TS-TCAP exposure, as compared to the control group (*P*< 0.0001). Furthermore, prolonging the treatment duration from 3 to 5 minutes resulted in a more pronounced effect. Consequently, subjecting cancer cells to argon TS-TCAP for 5 minutes led to a substantial reduction in CCSCs’ survival rate when compared to the control group. Similarly, parts (d), (e), and (f) are associated with the treatment effects of CCSCs using helium TS-TCAP for 3 and 5 minutes, respectively. The results indicated a significant decrease in the survival rate of CCSCs when exposed to helium TS-TCAP in comparison to the control group (*P*< 0.0001). Additionally, extending the treatment duration from 3 to 5 minutes exhibited a more significant impact. Hence, subjecting cancer cells to helium TS-TCAP for 5 minutes resulted in a marked decrease in CCSCs’ survival rate compared to the control group.

## Discussion

 In this study, our primary goal was to investigate the induction of apoptosis in CCSCs using TS-TCAP. This novel CAP was chosen for its ability to effectively deliver plasma-induced agents to CCSCs. The TS-TCAP technique involves a sequential discharge process, comprising primary, secondary, and tertiary stages, utilizing injected argon and helium gases. The plasma filaments generated during these stages play a crucial role in delivering the RONSs to the CCSCs.^21^ A significant aspect of this study is the emphasis on safety considerations. The voltage drop observed at the end of the second copper wire, coupled with the calculation of output plasma jet power, demonstrates the commitment to mitigating potential hazards associated with plasma-based therapies.^42^ In the same vein, with output power values below 1 W, the TS-TCAP approach ensures safe usage in medical applications, enhancing its feasibility for future clinical translation. Furthermore, the identification of reactive species through optical emission spectroscopy adds depth to the understanding of the plasma composition. The diverse range of excited species observed in both argon and helium plasma jets underscores the complexity of the plasma environment and its potential impact on cellular responses.^40^ Therefore, apart from assessing the existence of apoptotic cells, an investigation was conducted into multiple factors, including interleukins 6 and 8, Bax and BCL-2 proteins, as well as the KI-67 antigen, all of which signify either stimulation or induction of apoptosis. Both IL-6 and IL-8 play pivotal roles in regulating cell proliferation, adhesion, metastasis, and invasion in CCSCs.^43^ Additionally, CAP exhibits anti-inflammatory properties by attenuating inflammatory factors such as interleukin 6 and 8.^44^ In support of the above finding, our study demonstrated a decrease in gene expression and levels of IL-6 and IL-8 in the experimental groups exposed to TS-TCAP compared to the control group (*P*< 0.0001) (Figure 3). These findings underscore the potential of IL-6 and IL-8 signaling as valuable therapeutic markers for assessing the impact of TS-TCAP on cells.^39^ Consequently, a cell proliferation assay was first done in this study. Morphological investigations revealed the presence of typical apoptotic cells in all groups exposed to TS-TCAP (Figure 6). However, no formal statistical analysis was conducted to compare the number of apoptotic cells across different groups. In further confirmation of the above observation, the use of double-stained CCSCs with Annexin V-FITC and PI to quantify apoptotic cells (as depicted in Figure 4) demonstrated a significant rise in the number of apoptotic cells in CCSCs treated with TS-TCAP in comparison with the control group (49.7% versus 6.7%), and this difference was statistically significant (*P*< 0.0001). Moreover, it’s important to mention that the gene expression levels of BCL-2 and BAX were identified as standard markers for the detection of apoptosis.^45^ Conversely, research indicates that CAP exerts a beneficial influence on the expression of BCL-2 and BAX genes.^46^ According to the same principles, as shown in Figure 5, targeting CCSCs with TS-TCAP led to a significant decrease in BCL-2 gene expression and a substantial increase in BAX gene expression in all groups in comparison with the control group (*P*< 0.0001). Additionally, the figure underscores a direct correlation between the duration of TS-TCAP exposure and the extent of changes in gene expression. More precisely, the gene expression levels in the 5-minute exposure are higher than those in the 3-minute exposure for both helium and argon radiation. In support of the above finding, the ratio between BAX and BCL-2 expression, indicating a cell death switch, determines the survival or death of cells in response to stimulators of apoptosis, leading to enhanced cell death and reduced tumor incidence.^47,48^ It is very important to increase the expression of BAX/BCL-2 genes in CCSCs.^49^ Cell proliferation was assessed through the examination of the gene expression levels of KI-67. The detection of cellular proliferation by the gene expression of KI-67 is well known.^50,51^ Our study reveals a reduction in KI-67 expression in the treated groups (Figure 7), which is strongly correlated with a decrease in the number of CCSCs (*P* < 0.0001). This reduction in CCSCs could potentially result in a decrease in tumor growth. There are important findings indicating the anti-cancer potential of TS-TCAP. The results of the MTT assay reveal a decrease in the survival rate of CCSCs in all study groups in comparison to the control group, as demonstrated in Figure 8.

 Advancements in cancer diagnosis and treatment owe much to innovative technologies spanning diverse fields of science and engineering. The study affirms the success of TS-TCAT in effectively eliminating or deactivating CCSCs. Additionally, the presence of TS-TCAT underscores the significant role of RONS in eradicating CCSCs. Notably, even with a brief duration of 3 to 5 minutes, CAP treatment proved effective, inducing more damage to CCSCs compared to control cells during plasma treatment.^10^ The intricate interaction between CAP and living tissues poses challenges in comprehending the in vivo anticancer mechanisms of CAP.^52^ Reactive species, particularly reactive oxygen species, have been acknowledged as key contributors to cellular damage and eventual cell death in in vitro studies.^53,54^ CAP demonstrates a more pronounced induction of immune cell death compared to alternative methods. For instance, injecting CAP-treated CT26 CRC cells into mice resulted in a noticeable inhibition of tumor growth compared to the injection of untreated CT26 cells.^55^

 Research reveals that non-equilibrium atmospheric pressure plasma (NEAPP) generated by the plasma beam affects cancer-initiating cells (CICs). Tumorigenic cells, identified as CICs or CSCs, responsible for relapse and metastasis, were shown to exhibit elevated levels of aldehyde dehydrogenase expression.^56^ Investigating the impact of NEAPP on CIC using human endometrial adenocarcinoma cells and poorly differentiated human gastric cancer cells indicated its effectiveness on both CIC and non-CIC cells, influencing apoptosis.^56^ A synergistic effect of low-temperature plasma, combined with nanoparticle incorporation, was observed in human CRC (HCT-116). Treatment with gold nanoparticles followed by plasma irradiation led to an increased apoptosis rate in HCT-116 cells, although the exact mechanism remains unclear.^57^

 CAP technology demonstrated a significant reduction in the number of colon cancer cells and, intriguingly, the complete inactivation of these cells, as evidenced by an unchanged cell index after 16 hours, suggesting irreversible inactivation.^58^ The survival of colon cancer cell lines was considerably decreased with CAP treatment, indicating CAP’s selective antitumor impacts. Ongoing research explores the underlying mechanisms that give CAP its heightened potential against cancer cells compared to non-malignant cells and the differences in susceptibility to CAP among these cells.^59^ Findings emphasize the selective effect of CAP on cancer cells, a critical aspect of cancer therapy. Moreover, CAP treatment influences gene expression and methylation levels of significant factors in CRC.^60^

 In a specific study, plasma emerged as an effective inducer, capable of independent or combined use with other immunological approaches to trigger tumor-specific T-cell responses. Advancements in plasma transmission-based systems and administration protocols could position transferred plasmas as an independent or adjunctive, in vivo viable treatment modality for CRC immunotherapy.^33^ The research indicates that CAP affects both colon stem cells and colon epithelial cells, leading to tumor cell death, without any adverse effects on the healthy cells.^61^ Furthermore, combining CAP with vaccination enhances the specific T-cell response against cancer. In summary, immune cell death emerges as a crucial process in understanding the in vivo antitumor efficacy of CAP treatments.^33,55^

## Conclusion

 The results of this study show that using TS-TCAP with argon and helium gases can increase the expression of genes involved in CRC and also increase the rate of apoptosis. The study also found that the type of gas used and the duration of exposure play a significant role in influencing gene expression and cellular changes. In summary, the study demonstrates the effect of TS-TCAP on the occurrence of CCSC apoptosis in 3D culture media. The findings support the hypothesis that TS-TCAP is a novel biological tool with potential therapeutic applications that can affect cell structure and function and induce apoptosis. Importantly, TS-TCAP can be used as an alternative to chemotherapy or radiation to treat CCSC. Nevertheless, there are significant prerequisites and challenges in the way of this potential treatment approach. Conducting thorough pre-clinical and clinical trials, meticulously scrutinizing the impacts of CAP on all pertinent biological factors, devising safe delivery methods into the body, comprehensively assessing the risks and potential adverse effects associated with CAP, and investigating its synergistic effects with other treatment modalities are imperative. Evaluating the efficacy and safety of CAP in tumors resistant to current therapies through animal and human models represents one of these essential prerequisites. Detailed investigations and evaluations can potentially transform CAP into a nearly comprehensive and unparalleled treatment solution.

## Acknowledgments

 The authors received no financial support for the authorship and/or publication of this article.

## Competing Interests

 The authors declare no conflict of interest.

## Ethical Approval

 The methods, procedures, and experiments for cell handling received approval from the Ethics Committee of Tabriz University of Medical Sciences.
